# Supplementary material for: Attenuation of Pulmonary Fibrosis by the MyD88 Inhibitor TJ-M2010-5 Through Autophagy Induction in Mice
Source: Biomedicines. 2025 Sep 10;13(9):2214. doi: 10.3390/biomedicines13092214 (PMC12466944; doi:10.3390/biomedicines13092214)
Supplement: Supplementary file 1 [file biomedicines-13-02214-s001.zip › biomedicines-3821240-supplementary.pdf]

Supplementary Material

Supplementary Figures and Tables

1.1 Supplementary Table1

Detailed specific mouse primer sequences used in RT-PCR assays.

Gene	Forward sequence (5'→3')	Reverse sequence (5'→3')
MyD88	TTTATCTGCTACTGCCCAACG	GCGGCGACACCTTTTCT-CA
IL-1β	TGCCACCTTTTGACAGTGATG	TGTGCTGCTGCGAGATTTG
IL-6	AACAAGAAAGACAAAGCCAGAGT	ATTGGAAATTGGGGTAGGAAG
TNF-α	GTCTACTGAACTTCGGGGTGAT	TGCTACGACGTGGGCTACA
MCP-1	CCACTACCTTTTCCACAACCA	GGCATCACAGTCCGAGTCAC
TGF-β1	GCTGAACCAAGGAGACGGAATAC	CGTGGAGTTTGTTATCTTTGCTGTC
α-SMA	GCTTCGCTGGTGATGATGCTC	AGTTGGTGATGATGCCGTGTTC
FN	AATACGAAGTCAGTGTCTATGCTCTC	AGTGATTGTCTCTGTCTTTGTTCTCC
COL1A1	AAGGGACACCGAGGCTTCAG	CCAACAGCACCATCGTTACCG
GAPDH	TGTTCCTACCCCCAATGTGTCC	GGAGTTGCTGTTGAAGTCGCAG

1.2 Supplementary Figure S1

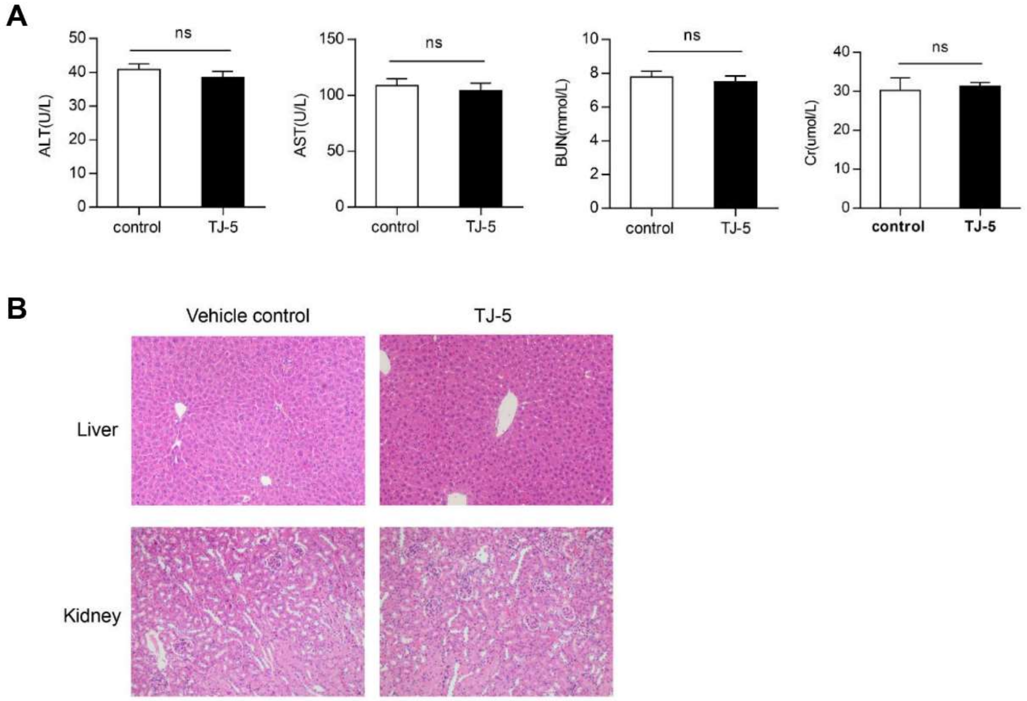

Supplementary Fig. S1. The effect of TJ-5 on the hepatic and renal functions and tissue morphology of mice. The mice treated with TJ-5 alone were intraperitoneally injected with TJ-5 (30 mg/kg) once a day for 21 days and mice in the control group were injected with an equal volume of ddH$_2$O. Blood samples and lung tissues were collected after 21 days. (A) Serum levels of ALT, AST, BUN, and Cr were measured (n = 6). ns, not significant. (B) Representative images of H&E staining of the liver and kidney. Scale bar: 50 µm. All data are presented as means ± SEM and are representative of three independent experiments.

1.3 Supplementary Figure S2

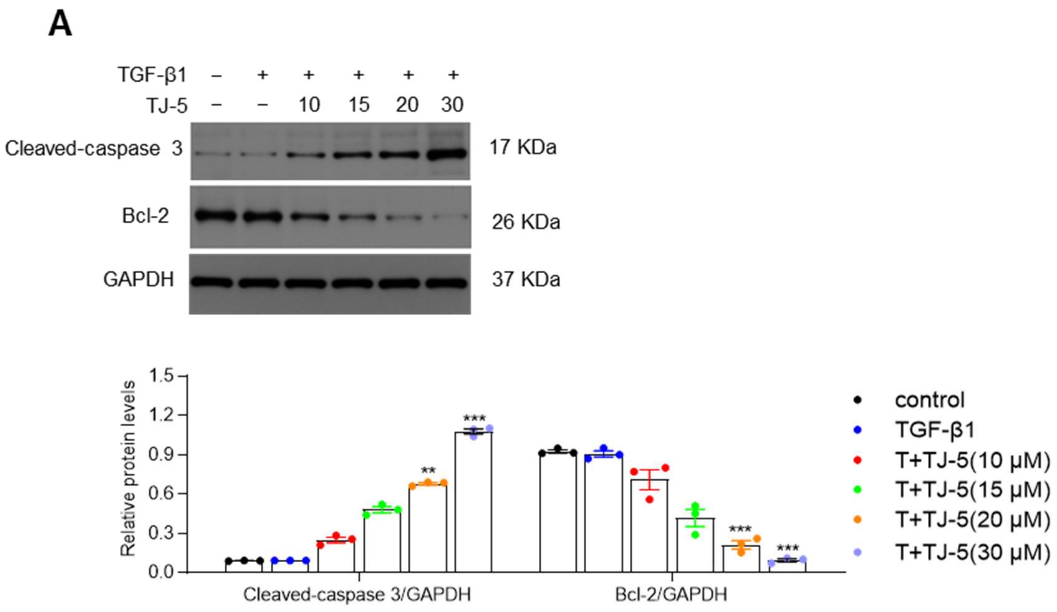

Supplementary Fig. S2. The effect of TJ-5 on the apoptosis of TGF-β1-induced MRC-5 cells. MRC-5 cells were pretreated with TJ-5 at varying concentrations (10 μM, 15 μM, 20 μM, 30 μM) for 2 h before being treated with TGF-β1 (5 ng/mL); 72 hours later, cells were harvested. T+TJ-5 indicates TGF-β1+TJ-5. (A) Representative western blot and quantitation of Cleaved-caspase-3 and Bcl-2 in MRC-5 cells (*n* = 3). **P < 0.01, ***P < 0.001 vs the TGF-β1 group. All data are presented as means ± SEM and are representative of three independent experiments.

Supplementary method:

Evaluation of hepatic and renal functions

Serum aspartate transaminase (ALT), alanine transaminase (AST), creatinine (Cr), and blood urea nitrogen (BUN) concentrations were detected using an automated biochemical analyzer BS-200 (Mindray, Shenzhen, China) to assess hepatic and renal function.
